# Protein Profile of Human Saliva as a Predictive and Prognostic Tool for OSCC in Tamol Chewer’s Population in Assam

**DOI:** 10.31557/APJCP.2021.22.6.1837

**Published:** 2021-06

**Authors:** Lhakit lepcha, Manash P Sarma, Amal C Kataki, Wankupar Wankhar, B G Unni

**Affiliations:** 1 *Department of Pathology , Assam Downtown University, India. *; 2 *Assam Down Town University Panikhaiti, Assam, India. *; 3 *Director Dr. Bhubaneswar Borooah Cancer Institute Guwahati, Assam, India. *

**Keywords:** OSCC, Oral squamous cell carcinoma, ANXA4, Annexin A4, HSP-Heat shock protein, SCC

## Abstract

**Objective:**

To identify potential proteomic salivary biomarker in tamol chewers and comparing it to healthy and Oral squamous cell carcinoma cases.

**Methods:**

A total of fifty unstimulated saliva samples were collected from the healthy volunteers, tamol chewers (without tobacco), and OSCC patients referred to North-East cancer Hospital, Jorabat, Assam, India. The 2-D gel analysis and western blotting were performed to analyze protein profiling.

**Results:**

The identified proteins were serum albumin, HSP (Heat shock protein) 27, gamma actin, SCC (Squamous cell carcinoma) 1, and Annexin A4. All the proteins were associated with OSCC development when their values were compared with those of normal healthy subjects. HSP27 was subjected to further validation using western blotting methods. An increase of 18.39% (Serum Albumin), 15.04% (gamma actin), 14.01% (SSC 1), and 20.22% (ANX4) were observed in Tamol chewers when compared with healthy control subjects.

**Conclusion:**

Our results revealed that the identified salivary proteins have a positive association with OSCC development. Profiling of these saliva proteomes especially HSP (Heat shock protein) 27 as a potential biomarker for OSCC detection in the high-risk population is recommended.

## Introduction

Oral cancer, histologically about 90% in the form of oral squamous cell carcinoma (OSCC), is a high-effect and common form of disease-related with the oral cavity (Gupta et al., 2013; Mascitti et al., 2018). Many environmental and genetic factors are often associated with the development of this disorder but the major regional predisposing risk factor for OSCC is chewing of betel quid and “paan” in India (Garg et al., 2014; Lepcha et al., 2021). World Health Organization and International Agency for cancer research classified areca nut as a group-I human carcinogen with enough verification of increased risk of precancerous oral lesion and cancer of the oral cavity (Hernandez et al., 2017). In Northeast India, a variety of raw areca nut is used in combination lime paste and piece of betel leaf without tobacco locally termed as tamol in Assam. Frequent and regular scratches of betel nut and betel leaf form ulcers in the oral cavity (Kumar et al., 2021). Comprises strong chemical compound in slaked lime which further forms scars or ulcers by burns the soft tissue in the oral cavity. Which can further be a main contributing factor for developing oral cancer (Phukan et al., 2001; Borkotoky et al., 2020). 

OSCC diagnosis depends on a thorough oral cavity examination, for possible signs and symptoms of the disease, followed by tissue biopsy. Besides diagnosis based on tissue, and body fluids like saliva and blood are the extensively studied samples that may comprise consistent biomarkers for cancer analysis. The saliva sample is an enlightening body fluid containing a range of analytes such as protein (Singh et al.,2020; Roi et al., 2020), mRNA (Oh et al., 2020), antioxidant profile (Lepcha et al., 2019), and DNA (Lepcha et al., 2021; Borkotoky et al., 2020) which can be used as diagnostic and prognostic markers for clinical and therapeutic applications. Several studies have investigated the use of salivary proteins as a potential diagnostic marker for oral cancer. The identification of proteins is either cleaved by gel electrophoresis or enzymatic digestion by the procedure to produce peptides (Hu et al., 2008; Ploypetch et al., 2020). Approximately, 3000 proteins have been identified in saliva by using various procedures (Hu et al., 2008; Jarai et al., 2012; Ploypetch et al., 2020). Similarly, various potential biomarkers identified from the saliva of OSCC such as cytokeratin 19 fragment (Cyfra21–1) (Rathore et al., 2020), albumin (Nguyen et al., 2020), telomerase (Sannam et al., 2016), transferrin (Nguyen et al., 2020), glutathione (Singh et al., 2020) were identified.

Thus, analyzing salivary protein profiles as potential biomarkers or molecular targets for early detection for OSCCs in tamol chewers and a feasible target to screen the population.

## Materials and Methods

*Methods*

*Sample preparation*

50 unstimulated saliva samples were collected from two groups of individuals i.e., raw betel nut chewers (non-alcohol and tobacco individuals), and control sample (non-raw betel nut, alcohol, and tobacco consumers) from Garo Goun, Mayang village, Panikhaiti area, and North-East cancer Hospital, Assam. Both males and females above 15 years were taken into consideration. All methods were performed in accordance with the relevant guidelines and regulations of the Ethics Committee of Dr. Bhubaneswar Borooah Cancer Institute, Assam, India (Registration no. BBCI/Misc-119/MEC/1065/2016). Protein was isolated by using Trizol solution and concentration of salivary protein was measured using the Bradford methods for tamol chewers and healthy individuals. 

*2-D electrophoresis*

Protein from tamol chewers and healthy individuals was dissolved in 125 µL of strip reswelling solution, soaked for a night at room temperature. And run in IEF unit, after isoelectric focussing strips were transferred into tray and covered with the reduction solution and incubate for 10 min. After 10 minutes, the reduction solution was removed and strips were covered with alkylation solution incubate for 10 mins. After 10 minutes alkylation solution was aspirated using Pipette and rinsed with tris glycine buffer. Strip was placed in SDS-PAGE gel (12%) above the resolving front and was overlayer with agarose heated at 70°C. the gels were stained with silver nitrate solution. The image was captured in gel documentation equipped with Canon EOS1300D.

*Western Blotting*

Western blotting was performed according to Towbin et al., (1984) Saliva samples were prepared with radioimmunoassay buffer (RIPA) (Sigma) and a protease inhibitor. Equal amounts of protein (30μg) were electrophoresis on 12 % SDS-PAGE. Following electrophoresis, separated proteins on SDS-PAGE gels were transferred onto the PVDF membrane (Millipore, USA). To block the nonspecific binding, membranes were incubated in blocking buffer with 5% skimmed milk for 2 h. Membranes were probed with primary antibodies (1:600) and blots were incubated with horseradish peroxidase-conjugated secondary antibodies (1:1,000). The bands were developed using an ECL kit (Millipore, USA) in the Chemi Doc image scanner from Bio-Rad. To estimate/quantify the band intensity, Quantity One software (Bio-Rad, USA) is used. The membranes were stripped and re-probed for β-actin (Sigma) (1:500) as an internal control.

## Results

*2D gel analysis of saliva sample in OSCC*

The demographic features and socio-economic position of the studied population are taken on a standard performa. In tamol chewers 23.6% of the studied population were male and 76.4% female respectively. Similarly, in the control sample 69% were male and 31% female. Higher proportions of females (76.4%) were found in the tamol chewer categories since the maximum male population was also involved in tobacco and alcohol consumption. No substantial difference was observed in both the categories concerning age, BMI, education, and profession.

**Figure 1:**
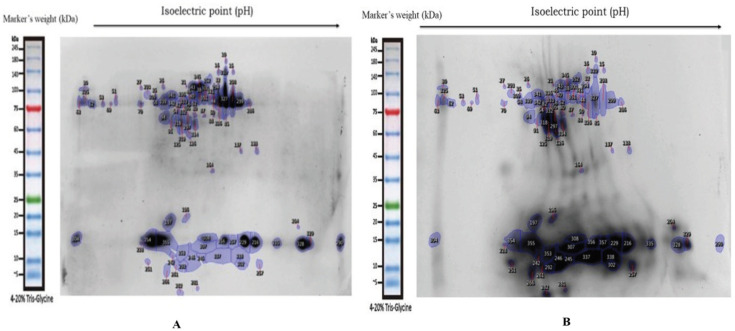
Image of 2D Gel Representing. A, Normal control; B, Tamol chewers sample

**Figure 2:**
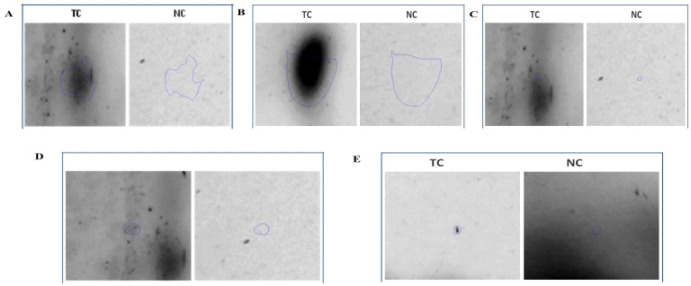
Five Upregulated Protein in Tamol Chewers as Compared with Normal Control. A, Serum albumin; B, Heat shock protein 1; C, Gamma actin; D, squamous cell carcinoma 1; E, ANXA4 while referring with their molecular weight (kDa)

The mean and SD of total protein of the control sample and tamol chewers were 1531.3 ± 256.1 and 4829.2 ± 2656.8 respectively. Proteins used for the 2-DE were evenly distributed in the 7 cm gel with pH the range from 3-10 and with molecular masses of 5-245kDa. Protein spots were detected in each gel by the Image Master software in Figure 1, which shows typical gel images for both normal control and tamol chewers population. 5 differentially expressed proteins had been identified and are upregulated or when compared with normal healthy individuals’ samples. Serum albumin, Heat shock protein 27 (HSP 27), gamma actin, squamous cell carcinoma (SCC) 1, and Annexin A4 (ANXA4) were identified proteins and have a positive association with OSCC development (Figures 1, 2 and Table 1).

*Protein expression of HSP27*

**Figure 3:**
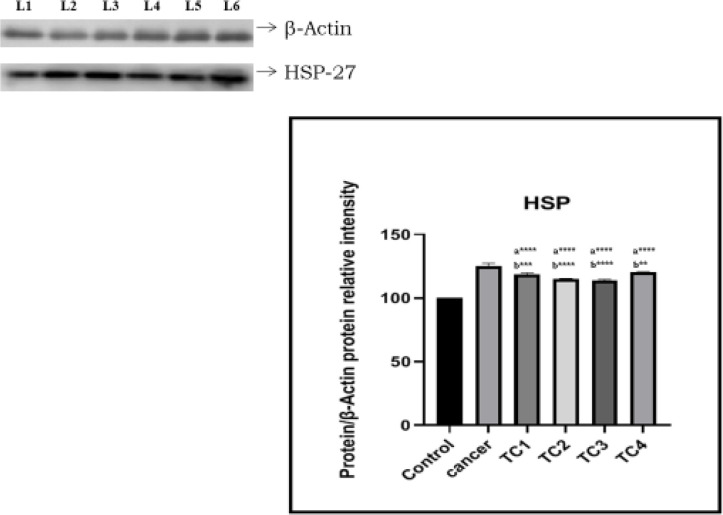
Protein Expression of HSP-27. L1: Con; L2: Cancer sample; L3: TC1 (Tamol chewers 1) - 1; L4: TC2 (Tamol chewers 2); L5: TC3 (Tamol chewers 3); L6: TC4 (Tamol chewers 4). Data are expressed as Mean ± SD. “a”- compared to control. “b” - Compared to Tamol chewers. The significance level was fixed at “*” p<0.05; “**” p<0.01; “***” p<0.001 and “****” p<0.0001

To understand the involvement of HSP27 protein, one control, one cancer sample, and four tamol chewers (TC) were run using western blotting technique. Band intensities in tamol chewers OSCC were all significantly higher with a consecutively upregulated trend from control and OSCC patient’s sample respectively. An increase of 18.39%, 15.04%, 14.01%, and 20.22% was observed in TC1, TC2, TC3, and TC4 when compared with the control sample respectively. Representative results were presented in Figure 3.

**Table 1:** List of Identified Proteins Found Upregulated in Tamol Chewers as Compared with Healthy Individuals

Spot number	Molecular weight (kDa)	Fold change	Protein name	Reference
318	67	2.9	Serum albumin	Koduru et al.,2017 Meurman et al.,2002
266	27	5.9	Heat shock protein 27	Lu et al.,2021 Wan et al., 2009 Zhu et al., 2010 Suzuki et al., 2007 Fan et al., 2020
257	41	3.8	Gamma Actin	Sannam et al., 2016 Duncan et al.,2008 Roman et al.,2013
196	45	5.2	Squamous cell carcinoma 1	Roman et al.,2013 Krapfenbauer et al.,2014 Nikitakis et al., 2003
204	34	3.8	ANXA4	Zimmermann et al., 2004 Gerke et al.,2005 Kim et al., 2010 Liu et al.,2016

## Discussion

Five different proteins identified were serum albumin, Heat shock protein 27, gamma actin, squamous cell carcinoma 1 (SCCA-1), and ANXA4 when compared to the control sample. These identified proteins have often shown a positive association with OSCC development either upregulated or . Family of actin are associated with cell mortality, junction organization, axon guidance, and also play important role in tumorigenesis. The presence of gamma actin in saliva can contribute especially to cancer diagnosis and prognosis (Sannam et al., 2016; Duncan et al., 2008; Roman et al., 2013). SCCA-1 another protein identified serves as an important tool for diagnosis and treatment marker for OSCC patients (Krapfenbauer et al., 2014). *SCCA-1* were found in tamol chewers then normal healthy case which indicates the positive association of tamol consumption with the development of OSCC. Similar results were reported as SCCA-1 as an important immunohistochemical marker in different cancer stages (Nikitakis et al., 2003). Upregulated SCCA-1 found in clinical patients with tongue cancer has been suggested as a potential biomarker not only for the tongue but also for Head and neck cancer (Roman et al., 2013).

Among all the annexins, ANXA4 is associated with cell adhesion, apoptosis, carcinogenesis, and invasion of cancer cells (Zimmermann et al., 2004). *ANXA4* was found in various other complications such as renal (Zimmermann et al., 2004), gastric cancer (Gerke et al., 2005), ovary cancer (Kim et al., 2010), and buccal squamous cell carcinoma (Liu et al., 2016). protein-enhanced significantly with tumor stage led to poor prognosis and associated with metastasis (Gerke et al.,2005; Kim et al., 2010). Koduru et al., (2017) reported a significant increase of salivary albumin in oral cancer cases as compared with healthy individuals and chronic genperiodonities. The increased salivary albumin level in oral pre-malignant and oral malignancy cases compared with healthy individuals served as an early diagnosis and prognostic role for oral malignant cases (Meurman et al., 2002). On the other hand, HPSs, also known as stress protein is expressed in a wide variety of physiological and environmental stress. Mammalian HSPs are classified into 5 categories according to their molecular weight (Lu et al., 2021). The ATP-dependent chaperone families HSP70 and HSP27 are the most studied protein because of their involvement in oral cancer (Lu et al., 2021) and are frequently related to the progression of tongue squamous cell carcinoma and also serve as an important biomarker (Wan et al., 2009). Our result has also identified the presence of HSP27 in oral squamous cell carcinoma in tamol chewers.

To confirm the involvement of HSP27, western blotting technique was carried out. *HSP27* expression was significantly higher in tamol chewers as compared with the control sample. An increase of 18.39%, 15.04%, 14.01%, and 20.22% was observed in TC1, TC2, TC3, and TC4 when compared with the control sample respectively. This supports the role of HSP27 in the development of oral cancer among tamol chewers population and potential salivary biomarker in oral squamous cell carcinoma. A similar result was stated by using proteomic technologies, among 85 different altered protein in OSCC, HSP27 was found upregulated in different histological grade of the premalignant lesion and OSCC (Wan et al., 2009; Zhu et al., 2010; Suzuki et al., 2007; Fan et al., 2020). 

In conclusion, our results reveal that profiling of saliva proteome can be a feasible marker to screen the population with the highest risk and also identify HSP27 as a potential salivary marker for OSCC detection. 

*Credit authorship contribution statement*

Lhakit Lepcha: Conceptualization, Methodology, Validation, Investigation, Investigation, Writing - original draft. Manash P Sarma: Conceptualization, Writing - review & editing. Amal C Kataki: Writing - review & resources. Wankupar Wankhar: Investigation, resources. BG Unni: Resources.

## Author Contribution Statement

None.
